# Medical Journals

**Published:** 1845-10

**Authors:** 


Medical Journals.—*Southern Medical and Surgical Journal.*—This excellent periodical is published monthly, at Augusta, Georgia, each monthly number containing 64 well-printed octavo pages; terms $3,00 per annum. It is edited by Paul T. Eve, and J. P. Garvin, M. D., two of the professors in the Medical College of Georgia, located at Augusta. The numbers which we have received contain a due proportion of original communications, many of them of exceeding interest, and exhibiting much research and observation on the part of the contributors. The selected matter and reviews do much credit to the discrimination and good taste of the editors. The proper acknowledgement of the receipt of this valuable journal, has been delayed in consequence of its having arrived during our absence, and since our return it has been unavoidably crowded out of our limited space.

*The American Journal and Library of Dental Science.*—This is a Quarterly issued in the city of Baltimore, and is the organ of the American Society of Dental Surgeons. It is edited by

Chapin A. Harris, M.D., D.D.S., Edward Maynard, M.D., D.D.S., Amos Westcott, M. D., D.D.S. It consists of two parts; (separately paged, so that they may be bound separately,) the Journal and Library. The Journal department is well conducted, and contains much that is valuable to the surgeon dentist and general practitioner, and interesting to the general reader. This department of the work contains from 70 to 90 pages. The Quarterly No. contains in all about 160 pages.

From the Journal we learn that the Surgeon Dentists are well organized and recognized as a distinct profession, and their *art* they have elevated to a *science*. They are associated in a national society, and properly consider all as *quacks* who are not regularly educated and legalized by their society or institutions, or deserve and purpose so to be. There are now, we believe, two Colleges of Dental Surgery, one at Baltimore, and one at Cincinnati. Of the former, we have before us the *sixth* annual announcement. The Faculty are as follows:—Chapin A. Harris, M. D., Professor of Practical Dentistry; Thomas E. Bond, Jr., Professor of Special Pathology and Therapeutics; W. R. Handy. M. D., Prof. of Anatomy and Physiology; Professorship (vacant) of Dental Physiology and Pathology; Chapin A. Harris, M.D., Demonstrator of Mechanical Dentistry. Regular courses of lectures are given by each professor; the session commencing on the first Monday of November, and ending the last of February. Ticket fees $25,00 to each professor.

*The New Orleans Medical and Surgical Journal.*—This journal comes to us improved in appearance, and strengthened in its editorial force. For some time past there has been in circulation a prospectus for a medical journal, to be published at New Orleans, and to be called the Louisiana Medical and Surgical Journal. An arrangement has now been effected, by which the two are united, with the strength of the co-editors of both journals, as follows: Erasmus D. Fenner, M.D., one of the Physicians to the New Orleans Charity Hospital; A. Hester, M. D., one of the Surgeons to the N. O. Charity Hospital; John Harrison, M. D., Professor of Physiology and Pathology in the Medical College of Louisiana; and W. M. Carpenter, M.D., Professor of Therapeutics and Materia Medica in the same institution. We consider it one of the most valuable of our exchanges.

*St. Louis Medical and Surgical Journal.*—In the editorial department of this journal, V. J. Fourgeaud has been associated with the former editors. We have on former occasions sufficiently expressed our good opinion of this periodical, and the additional labors of Dr. Fourgeaud must add to its former merits.

